# Pivotal gut microbiota and microbial metabolites involved in the regulation of simple obesity

**DOI:** 10.3389/fmicb.2026.1881046

**Published:** 2026-07-13

**Authors:** Yidan Gao, Haojie Zhang, Ruiping Jiang, Liyun Qin, Guoping Lv

**Affiliations:** 1 College of Integrative Medicine, Hebei University of Chinese Medicine, Shijiazhuang, Hebei, China; 2 Microbiology Testing Institute, Shijiazhuang Center for Disease Control and Prevention, Shijiazhuang, China; 3 Hebei Key Laboratory of Intractable Pathogens, Shijiazhuang, China

**Keywords:** gut microbiota, microbial metabolites, molecular docking, network pharmacology, simple obesity

## Abstract

**Introduction:**

Obesity increases the risk of multiple chronic diseases and has become a global health challenge. The gut microbiota influences the physiological and pathological processes associated with simple obesity. Elucidating the interactions between gut microbiota and simple obesity provides valuable insights for the development of effective weight-loss strategies.

**Methods:**

This study employed network pharmacology to investigate the molecular mechanisms between gut microbiota and simple obesity. Microbial metabolite targets were acquired from the Similarity Ensemble Approach (SEA) and Swiss Target Prediction (STP) databases, gut microbiota-related regulatory targets were retrieved from the gutMGene database, and disease-related targets were collected from GeneCards and the Online Mendelian Inheritance in Man (OMIM) database. Intersection analysis was performed to identify overlapping targets, and a gut microbiota–microbial metabolites–targets (MMTS) network was constructed. Molecular docking analysis was subsequently conducted to verify the binding affinity between key metabolites and targets.

**Results:**

Gut microbiota and microbial metabolites regulate simple obesity primarily through eight distinct targets, including IL6, PPARG, NFKB1, TLR4, TLR2, CXCL8, PTGS2, and HNF4A. A total of 23 microbial metabolites were identified, and numerous gut bacterial species associated with the core targets were characterized. Strains from the genera *Bifidobacterium*, *Lactobacillus*, *Akkermansia*, *Eubacterium*, *Bacteroides*, *Parabacteroides*, *Clostridium*, *Roseburia*, and *Streptococcus* exhibited significant regulatory effects in simple obesity.

**Discussion:**

These findings provide insights for understanding the role of gut microbiota and microbial metabolites in the regulation of simple obesity and provide scientific support for probiotics and metabolite-based interventions.

## 1 Introduction

Obesity is a major public health challenge of the 21st century. By 2030, 57.8% of the global adult population is estimated to be obese, while in China, 50.7% of Chinese adults were classified as overweight or obese in 2022 (Guo et al., 2024; Israel et al., 2022). More than half of the total population is affected by overweight or obesity, and the prevalence among children is more than 20% (Zhang et al., 2024). Obesity is associated with numerous chronic diseases, including type 2 diabetes, cardiovascular diseases, hypertension, dyslipidemia, and metabolic syndrome (Law et al., 2022; Sasso et al., 2023). Globally, the prevalence of obesity-induced chronic diseases continues to rise, and these conditions not only impose severe health impacts and heavy economic burden on society but also create formidable fiscal and public well-being challenges to humanity. Obesity results from the complex interplay of genetic, dietary, and behavioral factors. Traditionally, energy imbalance has been regarded as the core mechanism, with genetic factors determining susceptibility and sedentary lifestyle plus high-fat diet acting as major contributors (Masood and Moorthy, 2023). Beyond these established factors, emerging evidence suggests that additional key mechanisms are involved in obesity, including gut microbiota–gut–brain axis dysfunction, neuroendocrine dysfunction, chronic metabolic inflammation, epigenetic changes, psychobehavioral factors, and medication-induced weight gain (Młynarska et al., 2025; Singh et al., 2025). The gut microbiota and its microbial metabolites play important roles in regulating energy metabolism, inflammation, insulin resistance, appetite control, and lipid storage (Al Qassab et al., 2026; Borrego-Ruiz and Borrego, 2025). Therefore, investigations into the gut microbiota will provide novel perspectives for understanding the pathogenesis and therapeutic strategies of obesity.

The composition and structure of the gut microbiota community differ significantly between obese and non-obese individuals, with obese individuals showing a higher *Bacillota*-to-*Bacteroidota* ratio than non-obese individuals (Bressa et al., 2025). With the exacerbation of obesity, the abundance of beneficial bacteria, including *Faecalibacterium*, *Roseburia*, and *Ruminococcus*, gradually decreases, whereas potentially pathogenic bacteria, such as *Blautia*, *Collinsella*, and *Streptococcus*, tend to increase (Li E. et al., 2025). Gut-derived probiotics have been used as dietary supplements for the management of obesity in obese patients (Ali et al., 2025). The microbial metabolites, including short-chain fatty acids (SCFAs), bile acids (BAs), and tryptophan (Trp), can regulate obesity by affecting appetite, lipid metabolism, inflammatory responses and intestinal barrier integrity (Aghara et al., 2025; Agus et al., 2021). SCFAs play a major role in the alleviation of obesity: They can enhance the function of the intestinal barrier and reduce metabolic inflammation (Luo et al., 2025), regulate energy consumption by the modulation of host gene expression (Zachos et al., 2024), and inhibit the formation of fat by decreasing lipid accumulation (Hao et al., 2024). Identifying obesity-associated gut microbiota and metabolites and exploring their molecular mechanisms may provide insights for the development of simple obesity intervention studies.

Network pharmacology is a systematic analytical approach based on the interaction networks among diseases, genes, and protein targets, offers technical advantages such as multi-dimensional integrated analysis and molecular interaction network construction, overcomes the limitations of traditional single-target research, and enables systematic elucidation of the complex underlying mechanisms through which gut microbiota and its metabolites regulate diseases. This study uses approaches such as multi-database analysis, bioinformatics analysis and molecular docking to systematically analyze the molecular mechanisms underlying the regulation of simple obesity by gut microbiota and its metabolites. Through network pharmacology analysis, the study identified simple obesity associated bacterial species from gut microbiota and determined the key microbial metabolites. These findings not only provide theoretical support for the important regulatory role of gut microbiota in obesity but also lay the foundation for the development of weight loss-related probiotics and drugs.

## 2 Materials and methods

### 2.1 Identification of microbial metabolites and gut microbiota-related targets

The gut microbiota, microbial metabolites, and their associated targets were retrieved from the GutMGene database. The microbial metabolites were uploaded to the PubChem database to obtain their corresponding simplified molecular-input line-entry system (SMILES) format. Targets of microbial metabolites were predicted using the SEA and STP databases, with the species set as *Homo sapiens*, and a Venn diagram was adopted to integrate and intersect the targets obtained from the two databases. For the STP database, human targets with a prediction probability of ≥0.5 were retained. All data were sourced from database versions released before January 2026.

### 2.2 Identification of disease targets

Simple obesity-related genes were collected from GeneCards and the OMIM database using the keyword “simple obesity.” For GeneCards, a relevance score of ≥10 was used as the screening threshold. Genes retrieved from the two databases were intersected to obtain overlapping targets, and duplication entries were eliminated to generate a set of genes associated with simple obesity. The gene set associated with simple obesity was intersected with gut metabolite targets, and the resulting overlapping genes were further cross-referenced against human gut microbiota targets retrieved from the gutMGene database to identify potential therapeutic targets of simple obesity related to microbial metabolites. All data were sourced from database versions released before January 2026.

### 2.3 PPI network construction and analysis

The overlapping targets were uploaded to the STRING database to construct a protein–protein interaction (PPI) network. Moreover, the analysis results were exported in TSV format and subsequently imported into Cytoscape 3.10.2 to construct a network. To identify the core targets through which microbial metabolites regulate simple obesity, a network centrality analysis was performed using the CytoNCA plugin in Cytoscape 3.10.2.

### 2.4 Gene ontology and Kyoto encyclopedia of genes and genomes enrichment analysis

The overlapping targets were submitted to the DAVID platform to perform Gene Ontology (GO) and Kyoto Encyclopedia of Genes and Genomes (KEGG) analyses. GO function includes three categories: biological process (BP), cellular component (CC), and molecular function (MF). KEGG enrichment analysis was performed to identify potential pathways. Significantly enriched terms were defined as those with an adjusted *p*-value of < 0.05 and a FDR of < 0.05.

### 2.5 The microbiota–microbial metabolites–targets network analysis

The microbiota–microbial metabolites–targets (MMTS) network was constructed based on the gut microbiota, microbial metabolites, and core targets. This network was subsequently visualized using Cytoscape 3.10.2.

### 2.6 Molecular docking of microbial metabolites and targets

Molecular docking was performed to reduce the likelihood of false-positive results from the network pharmacology analysis. The structures of the microbial metabolites were downloaded from the PubChem database and converted into SMILES using ChemDraw. The structure files of the central targets were downloaded from the Protein Data Bank (PDB) database and saved in PDB format. The core targets and microbial metabolites were imported into AutoDock Tools for pre-docking processing. Moreover, AutoDock Vina was used for docking. Some of the docking results were visualized and analyzed using PyMOL software and Discovery Studio 2019 Client software.

## 3 Results

### 3.1 Identification of targets of microbial metabolites intervening in simple obesity

**Figure 1:**
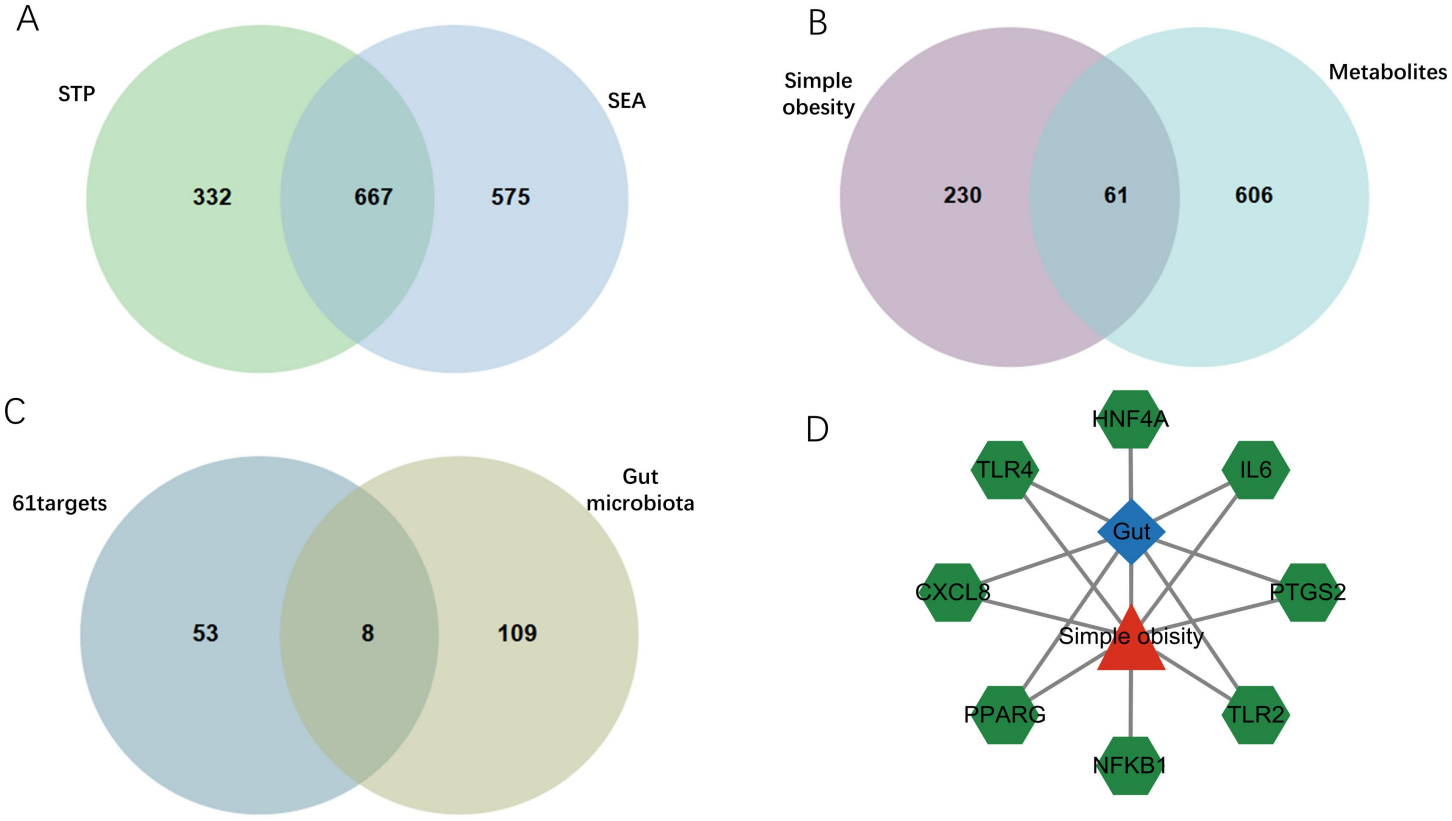
**(A)** The 667 microbial metabolite target genes. **(B)** The 61 overlapping genes between the 667 microbial metabolite targets and the 291 simple obesity genes. **(C)** The eight core targets between the 61 simple obesity-related microbial metabolite target genes and the 117 host genes. **(D)** The network of gut–target–simple obesity.

Data on 200 microbial metabolites and 117 targets of gut microbiota were retrieved from the gutMGene database. Targets of microbial metabolites were predicted using the SEA and STP databases, resulting in 1,242 and 999 targets, respectively. The 667 overlapping targets of microbial metabolites (Figure 1A) were considered the main targets of microbial metabolites. A total of 532 disease-related targets were retrieved from the GeneCards database, and 1,301 disease-related targets were retrieved from the OMIM database. The intersection of targets identified from both databases yielded 291 targets, which served as the common disease targets. The 61 targets are the intersection targets representing the overlap between the microbial metabolites and simple obesity (Figure 1B). An intersection analysis of the 61 metabolite targets and 117 gut microbiota-related targets yielded 8 overlapping targets (Figure 1C). The eight targets were regarded as the core targets that engaged in the development of simple obesity regulated by gut microbiota and its metabolites. On the basis of this, a gut–target–simple obesity network was constructed to elucidate the underlying complex relationships (Figure 1D).

### 3.2 Protein–protein interaction network analysis

**Figure 2:**
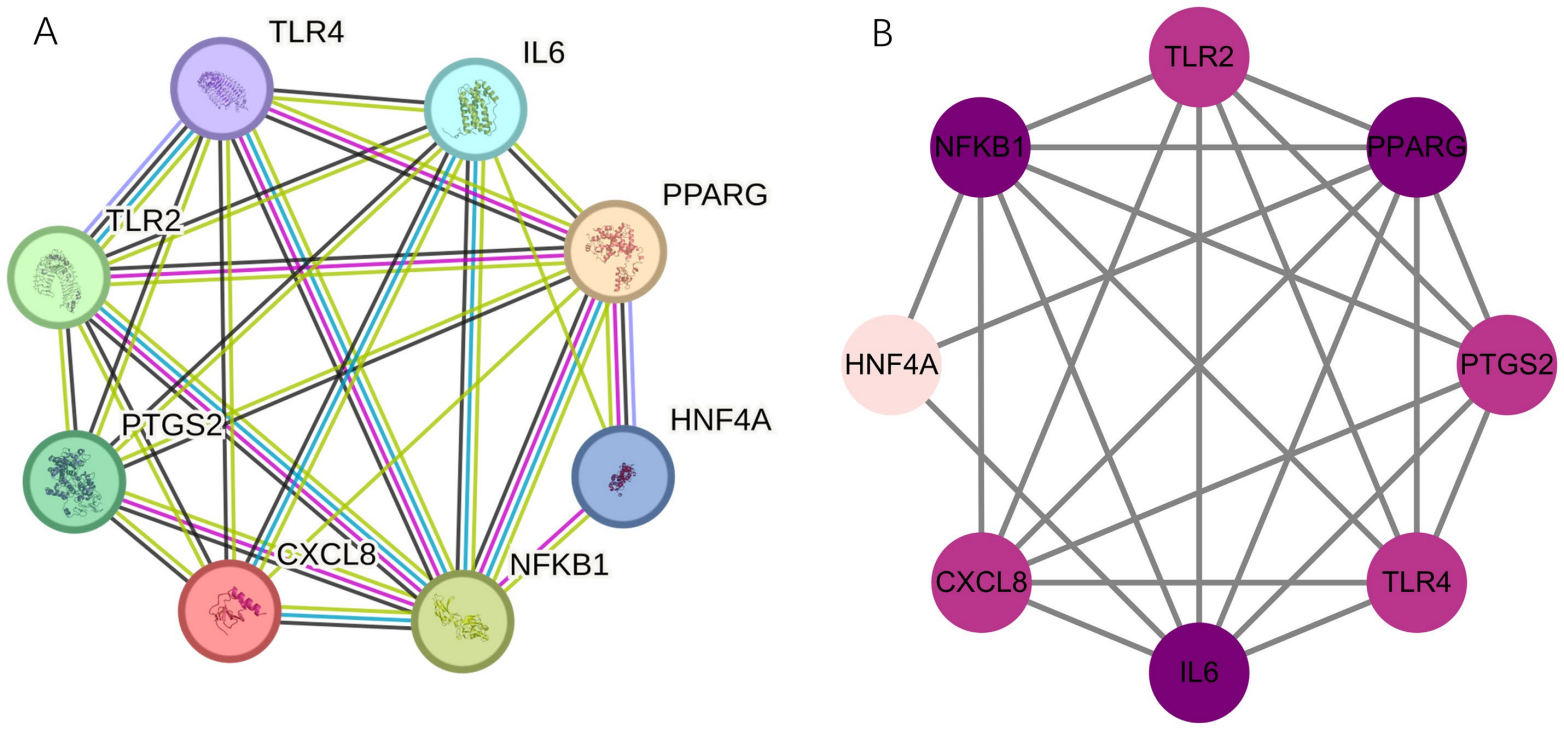
**(A)** The enrichment analysis of PPI network. **(B)** The visualization of PPI network.

To identify the core therapeutic targets for simple obesity treatment, PPI network analysis of the eight core targets was performed through the Search Tool for the Retrieval of Interacting Genes (STRING) platform (Figure 2A). The PPI network was visualized using Cytoscape 3.10.2 software and contained 8 nodes and 24 edges (Figure 2B). To further identify the core targets of the microbial metabolites in regulating simple obesity, an in-depth analysis of the centrality of the targets was performed using the Cytoscape plugin CytoNCA. The result revealed that IL6, PPARG, and NFKB1 were the most relevant therapeutic targets involved in the regulation of simple obesity by microbial metabolites based on their highest degree centrality (DC) values.

### 3.3 The GO and KEGG enrichment analysis

**Figure 3:**
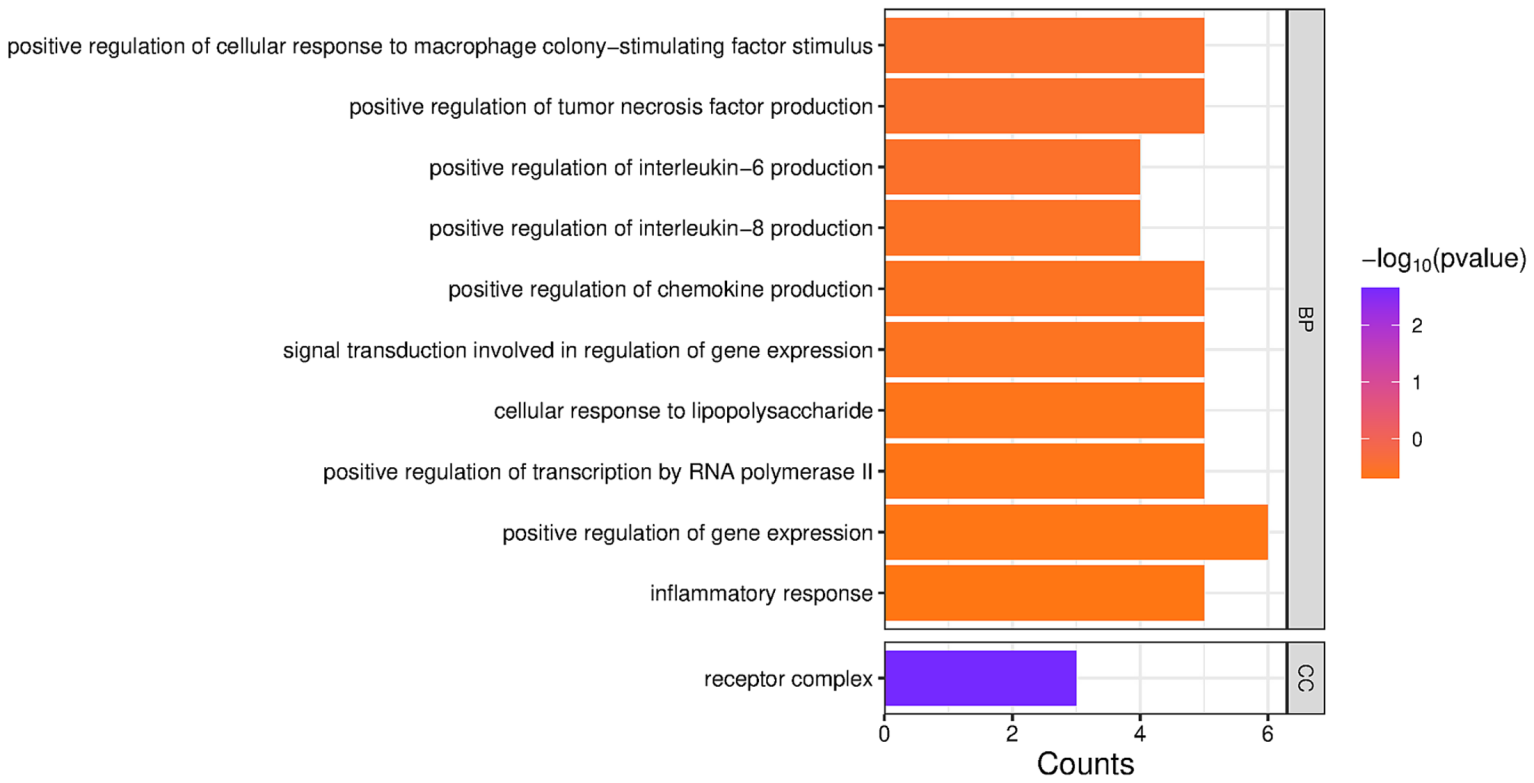
GO enrichment analysis.

**Figure 4:**
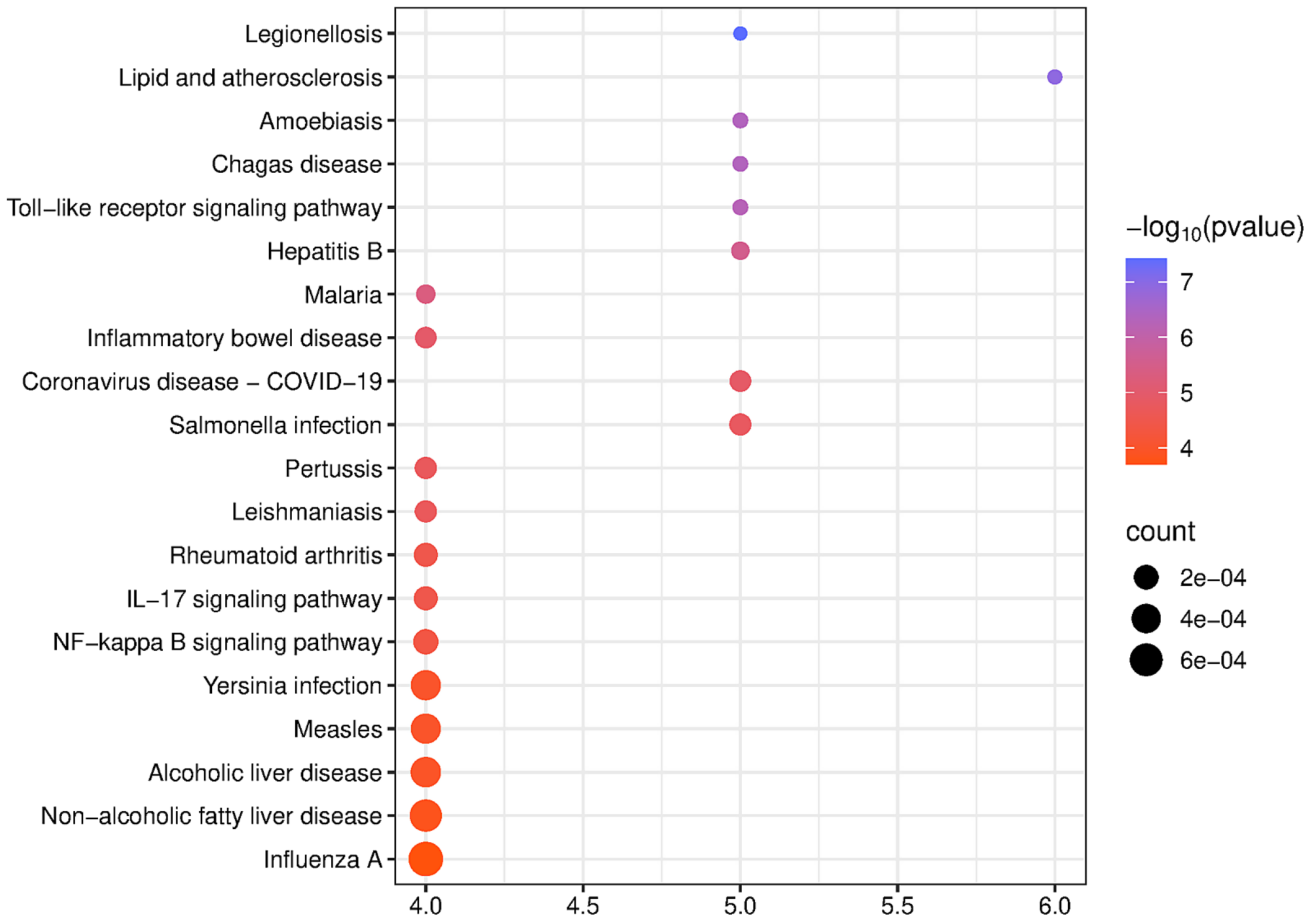
KEGG enrichment analysis.

To elucidate the biological functions of eight core targets in simple obesity regulation, an investigation of GO and KEGG enrichment analyses was conducted using the DAVID platform. Figure 3 reflects the core targets that regulate simple obesity. The most significantly enriched biological processes were inflammatory response, positive regulation of gene expression, and positive regulation of transcription by RNA polymerase II. The enriched cellular component terms are receptor complex and intracellular membrane-bounded organelle. For molecular functions, the top enriched terms include identical protein binding, lipopolysaccharide immune receptor activity, and transcription *cis*-regulatory region binding (Figure 3). In terms of KEGG pathways (Figure 4), the microbial metabolites exerting protective effects against simple obesity were primarily involved in the following major signaling pathways: inflammatory bowel disease, Toll-like receptor signaling pathway, and lipid and atherosclerosis.

### 3.4 The MMTS network

**Figure 5:**
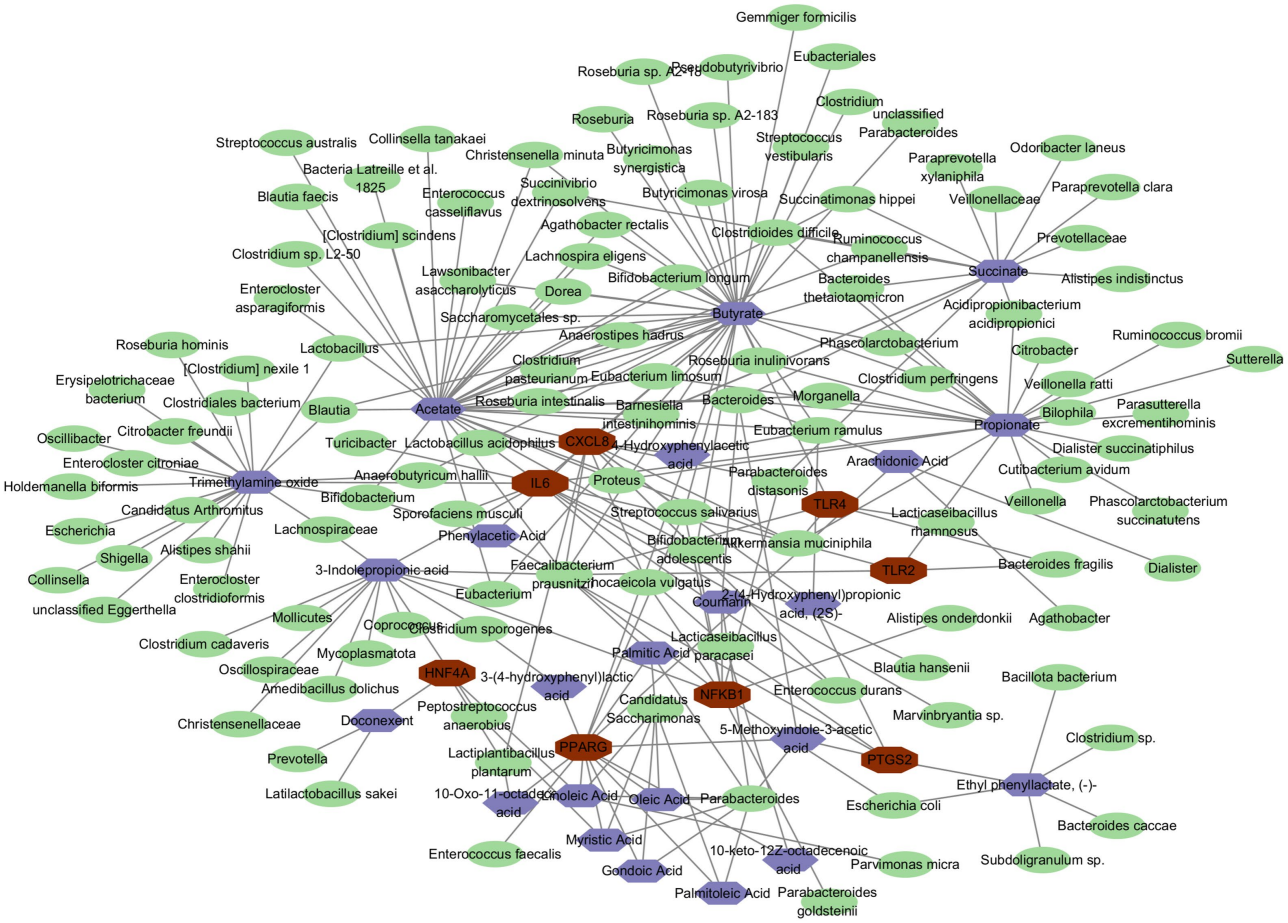
The network of gut microbiota–core target–microbial metabolite. The red nodes represent the eight core targets; the green nodes represent gut microbiota; the purple nodes represent microbial metabolites.

The MMTS network was constructed to elucidate intricate relationships among gut microbiota, microbial metabolites and core targets. The study identified 23 microbial metabolites and a number of gut microbiota associated with the eight core targets IL6, PPARG, NFKB1, CXCL8, TLR2, PTGS2, HNF4A, and TLR4. The interactions between these targets and their corresponding gut microbiotas and microbial metabolites were further explored. IL6 was regulated by five microbial metabolites (butyrate, acetate, propionate, 3-indolepropionic acid, trimethylamine oxide) and seven gut microbiotas (*Akkermansia muciniphila*, *Blautia hansenii*, *Enterococcus durans*, *Lacticaseibacillus paracasei*, *Marvinbryantia* sp., *Sporofaciens musculi*, *Turicibacter*). PPARG was associated with 12 microbial metabolites and 2 gut microbiota. The MMTS including gut microbiota, gut microbiota metabolites, and the core targets are shown in Figure 5. The strains involved in regulating simple obesity were primarily from the genera *Bifidobacterium*, *Lactobacillus*, *Akkermansia*, *Eubacterium*, *Bacteroides*, *Parabacteroides*, *Clostridium*, *Roseburia*, and *Streptococcus.*

### 3.5 The evaluation of drug-likeness and toxicity

A subsequent evaluation of the major metabolites’ drug-like properties (Table 1) and toxicity (Table 2) was performed. Based on the assessment of Lipinski’s Rule of Five, all evaluated metabolites conform to Lipinski’s Rule of Five with zero or only one violation, indicating a high probability of good oral bioavailability. Data on the Moriguchi LogP (MLogP), Lipinski’s violations, and bioavailability score for the metabolites arachidonic acid, linoleic acid, and doconexent are currently unavailable in the dataset. Toxicity testing results showed that arachidonic acid and coumarin exhibited potential carcinogenicity, whereas 5-methoxyindole-3-acetic acid and coumarin demonstrated Drug-Induced Liver Injury (DILI) positivity. Acetate, butyrate, propionate, and trimethylamine oxide showed Human Hepatotoxicity (H-HT) positivity. All microbial metabolites were human Ether-a-go-go Related Gene (hERG) non-blockers with Median Lethal Dose (LD50) ranging from 1.887 to 4.656 mg/kg.

**Table 1:** The physicochemical properties of the metabolites from key metabolites.

Compound	MW	HBA	HBD	MLog P	Lipinski’s violations	Bioavailability score	TPSA
Acetate	59.04	2	0	−0.49	0	0.85	40.13
Butyrate	87.1	2	0	0.49	0	0.85	40.13
Propionate	73.07	2	0	0.03	0	0.85	40.13
10-Oxo-11-octadecenoic acid	296.44	3	1	3.59	0	0.85	54.37
10-keto-12Z-octadecenoic acid	296.44	3	1	3.59	0	0.85	54.37
Arachidonic acid	304.47	2	1	–	–	–	37.3
5-methoxyindole-3-acetic acid	205.21	3	2	0.82	0	0.85	62.32
Gondoic acid	310.51	2	1	5.03	1	0.85	37.3
Linoleic acid	280.45	2	1	–	–	–	37.3
Myristic acid	228.37	2	1	3.69	0	0.85	37.3
Oleic acid	282.46	2	1	4.57	1	0.85	37.3
Palmitic acid	256.42	2	1	4.19	1	0.85	37.3
Palmitoleic acid	254.41	2	1	4.09	0	0.85	37.3
3-(4-hydroxyphenyl)lactic acid	182.17	4	3	0.52	0	0.56	77.76
Coumarin	146.14	2	0	1.65	0	0.55	30.21
Trimethylamine oxide	75.11	1	0	−1.66	0	0.55	29.43
3-indolepropionic acid	189.21	2	2	1.4	0	0.85	53.09
Succinate	116.07	4	0	−0.54	0	0.56	80.26
Phenylacetic acid	136.15	2	1	1.66	0	0.85	37.3
2-(4-hydroxyphenyl)propionic acid	166.17	3	2	1.37	0	0.85	57.53
4-Hydroxyphenylacetic acid	152.15	3	2	1.05	0	0.85	57.53
Ethyl phenyllactate, (−)−	194.23	3	1	1.7	0	0.55	46.53
Doconexent	328.49	2	1	–	–	–	37.3

**Table 2:** Toxicological properties of the metabolites from the key metabolites.

Metabolite	hERG blockers	H-HT	DILI	Carcinogenicity	LD50 (mg/kg)
Arachidonic acid	Non-blocker	Negative	Negative	Positive	3.966
5-methoxyindole-3-acetic acid	Non-blocker	Negative	Positive	Negative	4.485
Gondoic acid	Non-blocker	Negative	Negative	Negative	4.628
Linoleic acid	Non-blocker	Negative	Negative	Negative	4.153
Myristic acid	Non-blocker	Negative	Negative	Negative	4.127
Oleic acid	Non-blocker	Negative	Negative	Negative	4.393
Palmitic acid	Non-blocker	Negative	Negative	Negative	4.357
Palmitoleic acid	Non-blocker	Negative	Negative	Negative	4.165
3-(4-hydroxyphenyl)lactic acid	Non-blocker	Negative	Negative	Negative	3.948
10-keto-12Z-octadecenoic acid	Non-blocker	Negative	Negative	Negative	3.792
10-Oxo-11-octadecenoic acid	Non-blocker	Negative	Negative	Negative	4.553
Acetate	Non-blocker	Positive	Negative	Negative	2.597
Butyrate	Non-blocker	Positive	Negative	Negative	3.301
Propionate	Non-blocker	Positive	Negative	Negative	3.158
Coumarin	Non-blocker	Negative	Positive	Positive	4.656
Trimethylamine oxide	Non-blocker	Positive	Negative	Negative	1.887
3-Indolepropionic acid	Non-blocker	Negative	Negative	Negative	4.475
Succinate	Non-blocker	Positive	Negative	Negative	3.117
Phenylacetic acid	Non-blocker	Negative	Positive	Negative	3.244
2-(4-hydroxyphenyl)propionic acid	Non-blocker	Negative	Positive	Positive	3.816
4-hydroxyphenylacetic acid	Non-blocker	Negative	Positive	Negative	3.594
Ethyl phenyllactate, (−)−	Non-blocker	Negative	Negative	Negative	4.293
Doconexent	Non-blocker	Negative	Negative	Positive	3.801

### 3.6 Testing of microbial metabolites and targets for molecular docking

**Figure 6:**
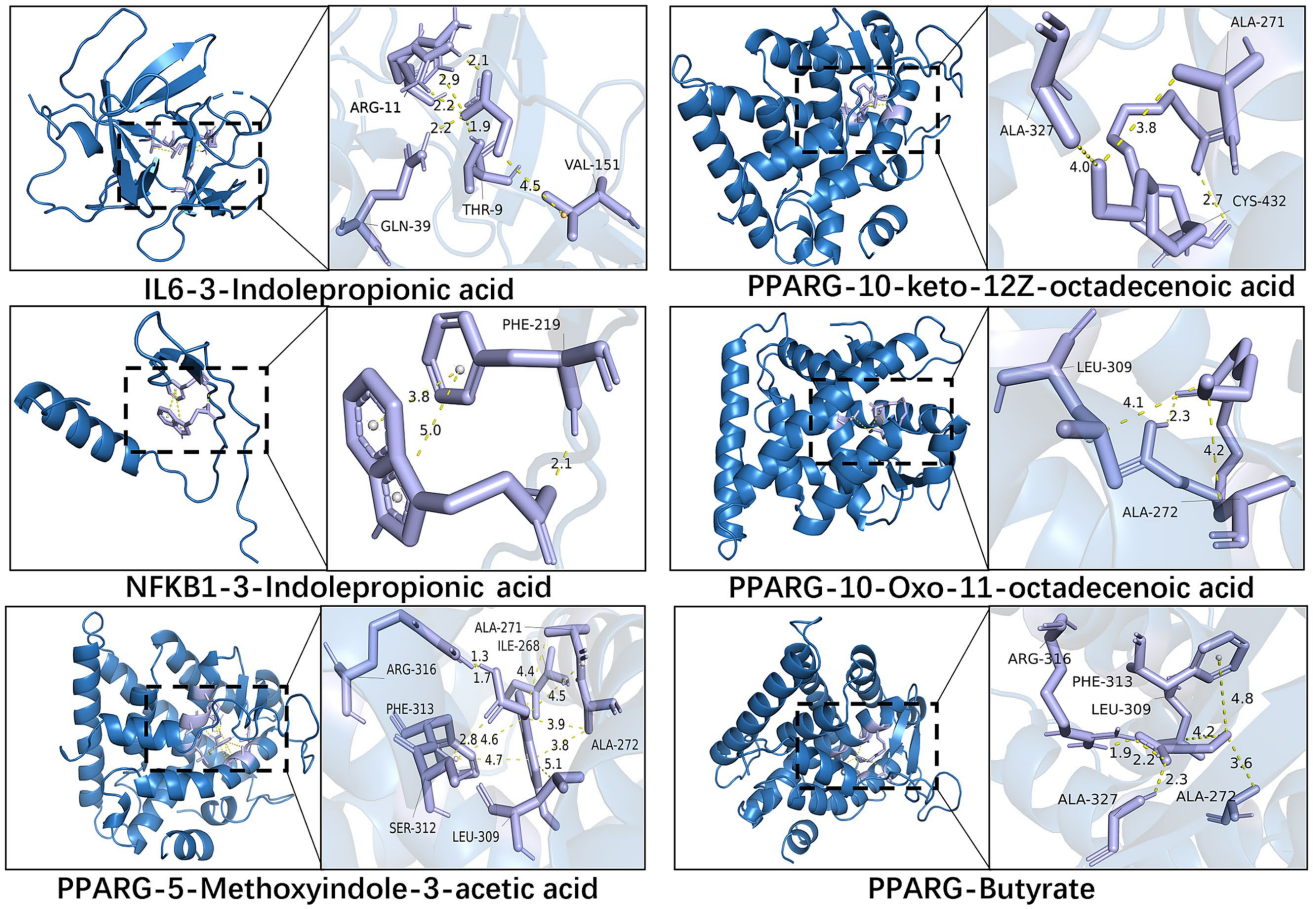
The molecular docking test on core target–metabolite pairs.

The binding affinities of the eight core targets (IL6, PPARG, NFKB1, CXCL8, PTGS2, TLR2, TLR4, and HNF4A) with 23 microbial metabolites were assessed. The molecular docking results showed that the binding affinity between the metabolites and targets ranged from −8.223 to −0.016 kcal/mol, all of which were below 0, suggesting spontaneous binding between the metabolites and targets. Among these, the docking results of the eight core targets with their corresponding metabolites are shown in detail in Table 3. The microbial metabolites exhibit good binding affinity with the core targets. A subset of complexes was selected for molecular docking and visualization, as shown in Figure 6.

**Table 3:** Molecular docking results of microbial metabolites and core targets.

Protein	Metabolite	Pubchem ID	Binding energy (kcal/mol)	Grid box center	Grid box dimension
IL6	Propionate	104745	−3.25	center_x = 38.703 center_y = 13.323 center_z = 68.395	size_x = 40 size_y = 40 size_z = 40
	Acetate	175	−2.972		
	Trimethylamine oxide	1145	−2.623		
	3-indolepropionic acid	3744	−5.322		
	Butyrate	104775	−3.351		
PPARG	5-methoxyindole-3-acetic acid	18986	−7.359	center_x = 6.99 center_y = −2.366 center_z = 42.83	
	Linoleic acid	5280450	−7.169		
	10-keto-12Z-octadecenoic acid	24970825	−6.913		
	10-Oxo-11-octadecenoic acid	10308378	−6.834		
	3-(4-hydroxyphenyl)lactic acid	9378	−6.694		
	Myristic acid	11005	−5.869		
	Arachidonic acid	444899	−5.541		
	Gondoic acid	5282768	−5.297		
	Oleic acid	445639	−4.684		
	Butyrate	104775	−4.447		
	Palmitoleic acid	445638	−4.326		
	Palmitic acid	985	−4.134		
NFKB1	Coumarin	323	−4.195	center_x = 18.749 center_y = 7.36 center_z = 3.644	
	3-indolepropionic acid	3744	−4.218		
CXCL8	2-(4-hydroxyphenyl)propionic acid	102526	−6.164	center_x = −9.054 center_y = 25.504 center_z = 33.249	
	4-hydroxyphenylacetic acid	127	−6.195		
	Phenylacetic acid	999	−5.356		
	Acetate	175	−2.718		
	Butyrate	104775	−3.579		
	Succinate	3693574	−0.016		
PTGS2	2-(4-hydroxyphenyl)propionic acid	102526	−7.605	center_x = 24.282 center_y = 44.482 center_z = 19.911	
	5-methoxyindole-3-acetic acid	18986	−8.223		
	Ethyl phenyllactate, (−)−	21115836	−7.283		
	Phenylacetic acid	999	−6.963		
TLR2	Propionate	104745	−3.29	center_x = −0.278 center_y = −12.003 center_z = −22.547	
TLR4	Butyrate	104775	−3.957	center_x = 10.472 center_y = −8.639 center_z = −8.102	
HNF4A	Doconexent	445580	−4.18	center_x = 67.642 center_y = 91.244 center_z = 9.039	
	Linoleic acid	5280450	−6.621		

## 4 Discussion

The gut microbiota exerts a significant impact on the physiological and pathological progression of simple obesity through bidirectional interactions along the gut–adipose axis (Liang et al., 2025). Gut microbiota dysbiosis impairs intestinal barrier integrity, induces chronic low-grade inflammation, promotes lipid synthesis and accumulation, and disrupts appetite homeostasis, thereby aggravating obesity, while a healthy gut microbiota improves these metabolic abnormalities and helps prevent and ameliorate obesity (Borrego-Ruiz and Borrego, 2025; Peña-Durán et al., 2025). Microbial metabolites exert anti-obesity effects by exerting anti-inflammatory actions, regulating lipid metabolism, promoting energy expenditure, suppressing adipogenesis and fat accumulation, and maintaining appetite homeostasis (Aghara et al., 2025; Du et al., 2025; Jiang et al., 2025). In this study, adopting a network pharmacology strategy, we systematically characterized the pivotal obesity-related targets modulated by gut microbiota and microbial metabolites, as well as the critical physiological and pathological processes associated with these targets.

A total of eight pivotal targets were identified by network pharmacology, including IL6, PPARG, NFKB1, TLR4, TLR2, CXCL8, PTGS2, and HNF4A. KEGG pathway enrichment analysis of these eight targets revealed that the gut microbiota and metabolites modulate simple obesity primarily via three core pathways: the Toll-like receptor signaling pathway, inflammatory bowel disease, and lipid and atherosclerosis. The top three targets in the MMTS network—PPARG, IL6, and NFKB1—are well-recognized core mediators of obesity pathogenesis. IL6 is markedly increased in obese individuals and metabolic syndrome patients, serving as a key marker of low-grade inflammation in obesity (Fic and Polak-Szczybyło, 2025; Mruczyk et al., 2025). Modulation of PPARG activity can prevent high-fat diet-induced obesity and insulin resistance (Sriboonaied et al., 2025). Aberrant activation of the NFKB signaling pathway in white adipose tissue is a hallmark of chronic low-grade inflammation in obese patients (Simao et al., 2024). The high rankings of these targets in the analysis of this study corroborate the mainstream consensus that inflammation and lipid metabolic disorders are the key pathological mechanisms of simple obesity. The target ranking of this study is largely consistent with the current understanding of obesity and also identifies additional regulatory nodes worthy of further exploration. TLR4 plays a vital role in obesity, inflammatory response, and glycolipid metabolic disorders (Alhamar et al., 2026). TLR2 expression is significantly increased in peripheral blood monocytes and adipose tissue of obese individuals (Wang et al., 2025). CXCL8 is secreted by macrophages in obese adipose tissue and exacerbates local inflammatory responses (He et al., 2024). HNF4A is a crucial nuclear receptor that controls hepatic carbohydrate and lipid metabolism, and it is closely associated with obesity (Dubois et al., 2024; Van Dender et al., 2026). PTGS2-encoded COX-2 disruption impairs metabolic homeostasis in diet-induced obesity (Balakrishnan et al., 2025). These core targets, combined with gut microbiota and microbial metabolites, exert synergistic effects and play crucial roles in the physiological and pathological processes of simple obesity.

In the MMTS network analysis, a variety of gut microbiota were identified. Among them, the core pivotal genera including *Bifidobacterium*, *Lactobacillus*, *Akkermansia*, *Eubacterium*, *Bacteroides*, *Parabacteroides*, *Clostridium*, *Roseburia*, and *Streptococcus* are closely involved in inflammatory signaling pathways and TLR4/NFκB signaling pathway by regulating IL1β, IL6, NFKB1, TLR4, TLR2, and CXCL8. *Clostridium* spp. alleviate endotoxemia and systemic inflammation, thereby ameliorating obesity-related metabolic disorders (Zhang et al., 2025). *Bacteroides* promotes intestinal homeostasis by regulating the expression mechanisms of HNF4A (Wang et al., 2024). Obesity leads to a decrease in *Akkermansia* and *Bacteroides*, as well as an increase in *Enterococcus*, which triggers metabolic endotoxemia, activates the TLR4/NF-κB pathway, and further elevates CXCL8 expression to induce adipose tissue inflammation (Yuan et al., 2021; Zhu et al., 2023). *Akkermansia muciniphila* helps alleviate obesity by strengthening the gut mucosal barrier (Wu et al., 2023), lowering blood lipid levels (Chanda and De, 2024), activating adipose tissue browning, enhancing thermogenesis, and improving energy expenditure efficiency (Zheng et al., 2023).

Furthermore, gut microbiota can synthesize bioactive metabolites, and these bioactive substances cooperate with intestinal bacteria to regulate the physiological and pathological progression of simple obesity through multi-target regulation. *Bifidobacterium* regulates simple obesity via the production and metabolism of SCFAs and simultaneously improves host energy metabolism and inflammatory status (Daneshpour et al., 2026; Lee et al., 2025). *Faecalibacterium*, *Agathobacter*, and *Roseburia* produce butyrate to reduce appetite, enhance intestinal barrier function, and alleviate endotoxemia and inflammation (Ito et al., 2023; van Deuren et al., 2022). *Parabacteroides* alleviates obesity and metabolic dysfunction by producing succinate and secondary BAs, enhances intestinal gluconeogenesis and barrier integrity, and improves host metabolism (Li Z. et al., 2025; Wang et al., 2019). *Lactobacillus* produces 10-Oxo-11-octadecenoic acid, which activates peroxisome proliferator-activated receptor gamma (PPARγ) to regulate energy metabolism and adipogenesis (Goto et al., 2015). In conclusion, the regulation of the physiological and pathological progression of simple obesity by gut microbiota is multifaceted and complex. Gut microbiota exerts regulatory effects either directly or through microbial metabolites. This study provides new insights into understanding the complex mechanisms through which gut microbiota modulates simple obesity.

In addition, a total of 23 differential metabolites were identified. SCFAs are involved in lipid synthesis and oxidation and alleviate obesity by regulating appetite, lipolysis, and thermogenesis (Münte and Hartmann, 2025; Zachos et al., 2024). Butyrate attenuates LPS-induced inflammation by inhibiting the TLR4/MyD88/NF-κB pathway, reduces the levels of IL-6, IL-8 (CXCL8), and TNF-α, upregulates PPARG expression, and facilitates lipid droplet formation and triglyceride accumulation (Liu et al., 2022; Sun et al., 2016). Propionate can promote the expression of genes related to gluconeogenesis, which helps reduce fat deposition (Apalowo et al., 2024). Acetate improves metabolism by enhancing fat oxidation and energy expenditure and markedly reduces IL-6 and TNF-α in LPS-stimulated monocytes of obese individuals (Eslick et al., 2022; González Hernández et al., 2020). 4-Hydroxyphenylacetic acid significantly slows weight gain in mice fed with a high-fat diet and ameliorates glucose intolerance as well as insulin resistance (Li Q. et al., 2025). Trimethylamine N-oxide is positively correlated with body mass index (BMI), visceral fat mass, and hepatic fat content and exacerbates insulin resistance (Barrea et al., 2019; Ilyas et al., 2022). 3-Indolepropionic acid restores colonic barrier integrity, markedly improves adipose tissue inflammation, prevents adipocyte hypertrophy, and ameliorates obesity (Chen et al., 2022; Ding et al., 2025). Docosahexaenoic acid ameliorates obesity-induced inflammation and insulin resistance (Molfino et al., 2016). Supplementation with docosahexaenoic acid can restore the elevated *Bacillota/Bacteroidota* ratio in the gut of obese children, increase the abundance of Bacteroidaceae, Oscillospiraceae, and Akkermansiaceae, and reduce the levels of Ruminococcaceae and Dialisteraceae (Lammi et al., 2025).

Gut microbiota and microbial metabolites are pivotal modulators of simple obesity. Microbiota-targeted interventions, including probiotics, prebiotics, and gut flora remodeling, can effectively ameliorate simple obesity. Probiotic supplements can reduce body weight and body mass BMI in overweight and obese individuals (Baek et al., 2025). Fecal microbiota transplantation (FMT) has become an emerging microbiota remodeling approach. FMT induces sustained changes in gut microbiome composition and functional potential, as well as improved fat distribution and insulin resistance in obese adolescents (Wilson et al., 2025). Developing precise nutritional strategies, tailoring interventions based on individual microbiota characteristics, selectively promoting beneficial microbiota, and optimizing SCFAs production represent a promising future direction for addressing obesity. In this study, the findings deepen the understanding of the microecological mechanism underlying obesity pathogenesis and provide novel theoretical references and actionable clues for clinical dietary regulation, intestinal microecological intervention, and the improvement of obesity-related metabolic disorders.

## 5 Study limitations and future prospects

This study has several limitations that should be acknowledged. First, the core targets and microbial metabolites were screened based on public databases, including GutMGene, GeneCards, OMIM, SEA, and STP. The incompleteness and delayed updating of these databases may introduce potential selection bias and affect the accuracy of the results. Second, network pharmacology is essentially a correlation-based prediction approach that cannot elucidate dose–response relationships or tissue-specific expression patterns of target genes, which limits the in-depth interpretation of molecular mechanisms. Third, the composition of gut microbiota varies significantly among individuals and populations due to genetic background, dietary habits, lifestyle and geographical factors, which may compromise the generalizability of the findings of this study.

Gut microbiota and metabolites affect simple obesity via multi-target and multi-pathway regulatory networks. The results of this study lay a foundation for understanding the molecular mechanisms underlying the regulation of simple obesity by gut microbiota and its metabolites. Moving forward, multi-omics approaches integrating metagenomics, transcriptomics, and metabolomics should be employed to comprehensively map the dynamic interactions between gut microbiota and host metabolism. Furthermore, *in vivo* validation using animal models of obesity and randomized controlled clinical trials are essential to confirm the causal relationships between the identified core targets, key bacterial strains, and obesity phenotypes. Ultimately, these efforts will facilitate the development of personalized microbiota-targeted interventions for the prevention and treatment of obesity.

## 6 Conclusion

This study systematically summarizes the core regulatory roles of gut microbiota and metabolites in simple obesity, characterized eight pivotal targets, and identified the main gut bacteria and 23 critical metabolites. The strains involved in regulating simple obesity were primarily from *Bifidobacterium*, *Lactobacillus*, *Akkermansia*, *Eubacterium*, *Bacteroides*, *Parabacteroides*, *Clostridium*, *Roseburia*, and *Streptococcus*. This research provides scientific evidence for the development of microecology-oriented prevention and treatment strategies against obesity and its related metabolic complications.

## Data Availability

The raw data supporting the conclusions of this article will be made available by the authors, without undue reservation.
